# **Preparation of g-C**_**3**_ **N**_**4**_ **-based photocatalytic nanofibers and antibiotic degradation performance research**

**DOI:** 10.1371/journal.pone.0333967

**Published:** 2025-10-10

**Authors:** Yanjin Wang, Zhuo Zhang, Lin Shi, Lidong Kou, Yasong Li

**Affiliations:** College of Environment Economics, Henan Finance University, Zhengzhou, Henan, China; University of Szeged, HUNGARY

## Abstract

With the extensive use of antibiotics around the world, their contamination problem has now attracted increasing attention, and seeking effective antibiotic contamination management technologies has been imminent. Visible light photocatalytic technology, as an emerging green technology with low cost, high efficiency and good application prospect, has become a research focus both at home and abroad. In this paper, graphitic phase carbon nitride (g-C_3_N_4_) was used as a photocatalytic material, and the composite photocatalysts g-C_3_N_4_/MoS_2_, g-C_3_N_4_/CuS, g-C_3_N_4_/CdS were produced by compounding it with a series of metal sulfides, and the physical and chemical properties of the composite photocatalysts were characterized by means of SEM, TEM, BET, XRD, XPS, FT-IR, and UV-VIS-NIR. The results show that the prepared composite photocatalytic materials do not change the morphological structure of g-C_3_N_4_, and the introduction of metal sulfides can effectively enhance the light absorption performance and photocatalytic activity of the composites. The best removal of typical antibiotic sulfadimethylpyrimidine (SMT) in water was achieved by g-C_3_N_4_/CdS, with the most stable photocatalytic performance, and the complete removal of SMT could be achieved after 6 h of photocatalysis under LED illumination conditions at wavelengths of 420 nm and 365 nm. g-C_3_N_4_/CdS had the highest photocatalytic activity at pH = 3. Optimal g-C_3_N_4_/CdS was used to prepare flexible polyacrylonitrile carrying carbon nitride nanofiber photocatalysts (PAN/g-C_3_N_4_/CdS) by electrostatic spinning method, which had excellent light absorption properties in UV and visible light regions. SMT removal of PAN/g-C_3_N_4_/CdS reached 100.00% after the light was turned on for 6 h at a wavelength of 365 nm, indicating that the nanofiber photocatalytic materials prepared in this study have excellent photocatalytic activity and antibiotic degradation performance. This paper has important theoretical and practical significance for solving the problem of antibiotic pollution in water bodies.

## Introduction

One of the most commonly used antibiotics, sulfonamide antibiotics have also been frequently detected in water environments in recent years [1]. Trace amounts of sulfonamide antibiotic residues in water environments can lead to the development of resistant bacteria and resistance genes, jeopardizing human health and ecological environment [2–4]. With the increase in the production and use of sulfonamide antibiotics, the water treatment problems arising therefrom have gradually attracted industry attention [5,6]. In the current water treatment process, it is generally focused on the conventional water quality indexes, but the removal of organic pollutants such as sulfonamide antibiotics is rarely reported [7,8]. Among the methods for degrading sulfonamide antibiotics, visible light photocatalytic degradation technology has become a popular research topic because of its mild reaction conditions and low energy consumption [9–13].

As an efficient and stable photocatalyst with stable chemical structure and response to visible light, g-C_3_N_4_ can effectively promote the degradation of sulfonamide antibiotics [14,15]. The photocatalyst suffers from problems such as easy complexation of photogenerated carriers, low mobility, and low utilization, which limit the improvement of its catalytic efficiency [16,17]. To overcome these difficulties, researchers have actively explored various optimization strategies, such as constructing suitable heterojunction structures, introducing noble metal doping, and performing structural modifications [18,19]. These strategies have enhanced the performance of photocatalysts to a certain extent, but still failed to fully break the bottlenecks in their practical applications, which are mainly the poor mechanical properties of photocatalysts and the difficulty of recycling and reuse [20–22]. To tackle these difficulties, an innovative solution is proposed in this paper, in which g-C_3_N_4_ is used as the photocatalytic material, and ammonium molybdate, copper nitrate and cadmium nitrate are milled and calcined with thiourea, respectively, to prepare graphite-phase carbon nitride/metal sulfide composite photocatalytic materials using a one-step thermal polymerization method [23]. The carbon nitride composite photocatalyst with optimal performance was then loaded onto PAN nanofibers by electrostatic spinning to prepare flexible polyacrylonitrile carrying carbon nitride nanofiber composites, to further study the removal performance of this photocatalyst for SMT [24–26]. This composite material includes the excellent performance of PAN nanofibers, and also significantly improves its photocatalytic activity through the introduction of carbon nitride, which provides new ideas and methods for more in-depth study of photocatalytic degradation of antibiotics, and is of great theoretical and practical significance for solving the problem of antibiotic contamination in water bodies.

## Experiment

### Experimental materials

Thiourea CH_4_N_2_S, Shanghai Aladdin Biochemical Technology Co., Ltd; Ammonium molybdate (NH_4_)_6_Mo_7_O_24_·4H_2_O, Tianjin Hengxin Chemical Industry Co., Ltd; Copper Nitrate Cu(NO_3_)_2_·3H_2_O, Zhengzhou Paini Chemical Reagent Factory; Cadmium Nitrate Cd(NO_3_)_2_·4H_2_O, Xilong Scientific Co., Ltd; Polyacrylonitrile PAN, Tianjin Kermel Chemical Reagent Co., Ltd; N,N Dimethylformamide C_3_H_7_NO, Yantai Shuangshuang Chemical Co., Ltd; Sulfadimethoxine C_12_H_14_N_4_O_2_S ≥ 99%, Shanghai Macklin Biochemical Technology Co., Ltd; Glacial Acetic Acid CH_3_COOH, Tianjin Fuyu Fine Chemical Co., Ltd; Sodium Hydroxide NaOH, Fuchen Chemical Reagent Co., Ltd; acetonitrile C_2_H_3_N ≥ 99.9%, Shanghai Macklin Biochemical Technology Co., Ltd; Potassium Bromide KBr, spectrally pure, Tianjin Kermel Chemical Reagent Co., Ltd; Hydrochloric Acid HCl, Yantai Shuangshuang Chemical Co., Ltd. All reagents used above are analytical grade.

### Experimental methods

#### Catalyst preparation method.

g-C_3_N_4_ photocatalytic materials were prepared by thermal polymerization using thiourea as a precursor [27,28]. 8 g of thiourea was weighed and placed in a crucible, and heated in a tubular furnace under N_2_ atmosphere to 500°C at a rate of 5°C/min, then held at 500°C for 2 h. After being naturally cooled to room temperature, the product was weighed and set aside, and the g-C_3_N_4_ photocatalytic material was thus obtained.

First, 0.175 g of cadmium nitrate was weighed and dissolved. The cadmium nitrate solution was slowly added to the thiourea crystals, and the mixture was dried in an oven at 80°C until a constant weight was achieved. The dried mixture was then ground into a fine powder and weighed. The ground mixture was placed in an alumina ceramic crucible and loaded into a tubular furnace, where it was heated under N_2_ atmosphere to 500°C at a rate of 5°C/min, then held at 500°C for 2 h. After naturally cooling to room temperature, the product was washed with water to remove impurities and dried to a constant weight, yielding the g-C_3_N_4_/CdS composite material. Under the same conditions, ammonium molybdate and copper nitrate were used to replace cadmium nitrate, producing g-C_3_N_4_/MoS_2_ and g-C_3_N_4_/CuS composite materials, respectively [29,30].

0.15 g of the as-prepared g-C_3_N_4_ powder was dissolved in 5 mL of N,N-dimethylformamide (DMF) and ultrasonicated for 1 h. Then, 0.75 g of polyacrylonitrile (PAN) was added to the mixture and magnetically stirred for 48 h until a homogeneous pale-yellow solution was obtained [31]. The PAN/g-C_3_N_4_ composite nanofibers were fabricated using an electrospinning machine (NANON-01A, MECC Co., Japan) under the following conditions: an applied voltage of 15 kV, a feeding rate of 0.4 mL/h, and a needle movement range of 0–150 mm. The resulting electrospun fibers were rinsed several times with distilled water and dried in an oven at 60°C, denoted as PAN/g-C_3_N_4_ composite nanofibers [32,33]. Similarly, 0.15 g of g-C_3_N_4_/CdS was processed under the same conditions to obtain PAN/g-C_3_N_4_/CdS nanofibers.

#### Characterization method.

The obtained materials were characterized and analyzed using a Fourier Fourier transform infrared spectrometer (INVENI0, Bruker (Beijing) Scientific Technology Co. Ltd.‌‌), a UV-NIR spectrophotometer (Cary7000, Agilent Technology (China) Co., Ltd.), a scanning electron microscope (S-4800, Hitachi, Japan), field emission transmission electron microscope (TEM, JEOL-JEM-F200, Japan), fully automated physical adsorption analyzer (BET, Micromeritics ASAP 2460, USA), X-ray polycrystalline diffraction (XRD, Rigaku SmartLab SE, Japan), and X-ray photoelectron spectroscopy (XPS, Thermo Fisher K-Alpha, USA).

#### Experiment on photocatalytic degradation of SMT.

10 mg of photocatalytic material was added to 50 mL of sulfadimethoxine solution at a concentration of 0.1 mmol/L. The obtained suspension was placed in a multi-channel photocatalytic reaction system (PCX-50C, Beijing Perfectlight Technology Co., Ltd.) and stirred at 300 r/min for 1 h in darkness at 28°C to achieve adsorption equilibrium between sulfadimethoxine and the photocatalytic material. Subsequently, the LED light source was turned on to initiate the degradation of sulfadimethoxine under either white light (5 W, wavelength range 380–760 nm) or monochromatic light (5 W, wavelengths 365 nm, 420 nm, 630 nm, or 880 nm) [34,35]. During the photocatalytic process, 1 mL of the reaction solution was sampled at predetermined intervals, filtered, and the change in SMT concentration in the filtrate was analyzed by high-performance liquid chromatography (Agilent-1260, Agilent Technology (China) Co., Ltd.) under the following conditions: 0.1% acetic acid: acetonitrile = 60%: 40%, flow rate: 1 mL/min, column temperature: 40°C, and detection wavelength: 264 nm. The SMT removal rate was calculated based on the concentration change using the following formula:


$$\eta =({\text{C}}_{\text{0}}-{\text{C}}_{\text{t}})/{\text{C}}_{\text{0}}\times \text{100}\%$$

Where C_0_ is the initial concentration of SMT solution, mM/L, C_t_ is the concentration of SMT solution at any time t, mM/L, η is the SMT removal rate.

## Results and analysis

### Characterization of photocatalytic materials

#### Structure and morphological analysis.

**Fig 1:**
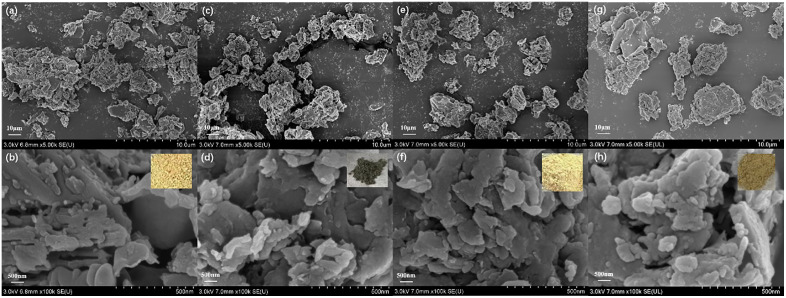
SEM images of carbon nitride composite material (the upper right corner: optical photograph) (a, b): g-C_3_N_4_, (c, d): g-C_3_N_4_/MoS_2_, (e, f): g-C_3_N_4_/CdS, (g, h): g-C_3_N_4_/CuS.

**Fig 2:**
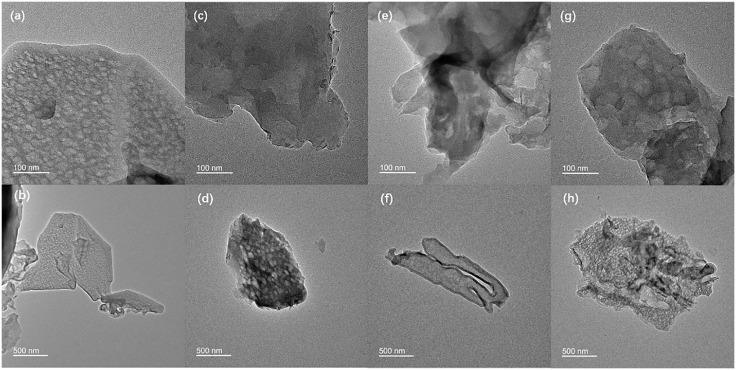
TEM images of carbon nitride composite material (a, b): g-C_3_N_4_, (c, d): g-C_3_N_4_/MoS_2_, (e, f): g-C_3_N_4_/CdS, (g, h): g-C_3_N_4_/CuS.

Fig 1 shows the SEM image of carbon nitride composite photocatalytic material with its optical photograph in the upper right corner of the figure. Fig 1a and 1b show the surface morphology of g-C_3_N_4_ at different magnifications, which has yellow powdery appearance. Under magnification of 5 K, g-C_3_N_4_ was observed as irregular particles; under magnification of 100 K, each particle was observed to be stacked by lamellar structure, with a size of 200–300 nm and a thickness of ﹤50 nm. Fig 1c and 1d are the surface topography of g-C_3_N_4_/MoS_2_ composites with dark gray powdery appearance, and Fig 1e and 1f are the surface topography of g-C_3_N_4_/CdS composites with light yellow powdery appearance. The difference in color of the two composite photocatalytic materials indicates that new and different substances were generated in the preparation, where MoS_2_ was black and CdS was lemon yellow, which led to the transformation of the corresponding composites to dark gray or to remain light yellow. Comparison shows that there is no obvious difference in the surface morphology and lamellar structure between the composite photocatalytic material and pure g-C_3_N_4_, indicating that the prepared composite photocatalytic material does not change the morphological structure of g-C_3_N_4_. Fig 2 shows the TEM image of the carbon nitride composite photocatalytic material. Analysis shows that g-C_3_N_4_ exhibits a porous layered structure with high crystallinity and abundant active sites. In the g-C_3_N_4_/MoS_2_ composite material, MoS_2_ nanosheets are uniformly distributed on the surface of g-C_3_N_4_, forming a tight heterojunction interface. The g-C_3_N_4_/CdS composite material shows that CdS nanoparticles are uniformly dispersed on the g-C_3_N_4_ layer. The heterojunction structure of composite materials significantly optimizes interface contact and band matching, providing a key structural foundation for improving photocatalytic performance.

**Fig 3:**
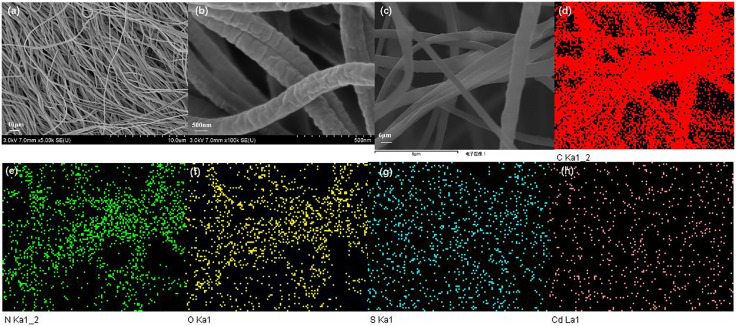
SEM images (a, b) and EDS mapping images (c, d, e, f, g, h) of PAN/g-C_3_N_4_/CdS.

Fig 3a and 3b shows SEM images of PAN/g-C_3_N_4_/CdS at magnification of 5 K and 100 K. The flexible fiber material is a fiber structure with rough surface, with diameter of 0.5 ~ 1 μm, and rough scale structure on the fiber surface, which is most likely caused by the addition of the lamellar structured g-C_3_N_4_/CdS composite photocatalyst in electrostatic spinning solution. Electrostatic spinning could contribute to the improvement of the agglomeration performance of photocatalysts, which made the photocatalyst lamellae uniformly dispersed in nanofiber structure. Fig 3(c ~ h) and Table 1 show EDS images, and it can be found that the weight percentages of S and Cd are 0.16% and 0.58%, respectively, and the elements of O, C, N, S and Cd are uniformly distributed on the surface of the samples, which proves that PAN/g-C_3_N_4_/CdS is successfully prepared.

**Table 1:** EDS atomic percentage of PAN/g-C_3_N_4_/CdS.

Element	Weight/%	Atomic/%
C	53.96	59.10
N	33.63	31.27
O	11.67	9.51
S	0.16	0.06
Cd	0.58	0.06

The specific surface area of carbon nitride composite materials is shown in Table 2. After the composite, the specific surface area of g-C_3_N_4_/MoS_2_ decreased to 3.6384 m^2^/g, and the specific surface area of g-C_3_N_4_/CdS increased to 6.1973 m^2^/g. The change in specific surface area was not significant, so the specific surface area was not the main factor affecting catalytic activity. However, the composite synergistically regulates the degree of pore and interface exposure, further optimizing catalytic performance.

**Table 2:** Specific surface area and pore structure of carbon nitride composite material.

Material	BET specific surface area/(m^2^/g)	Total pore Volume(cm^3^/g)	Average pore size (nm)
g-C_3_N_4_	5.9372	0.03	30.21
g-C_3_N_4_/MoS_2_	3.6384	0.02	40.33
g-C_3_N_4_/CdS	6.1973	0.04	25.28
g-C_3_N_4_/CuS	6.0215	0.04	23.29
PAN/g-C_3_N_4_/CdS	6.2058	0.04	26.33

**Fig 4:**
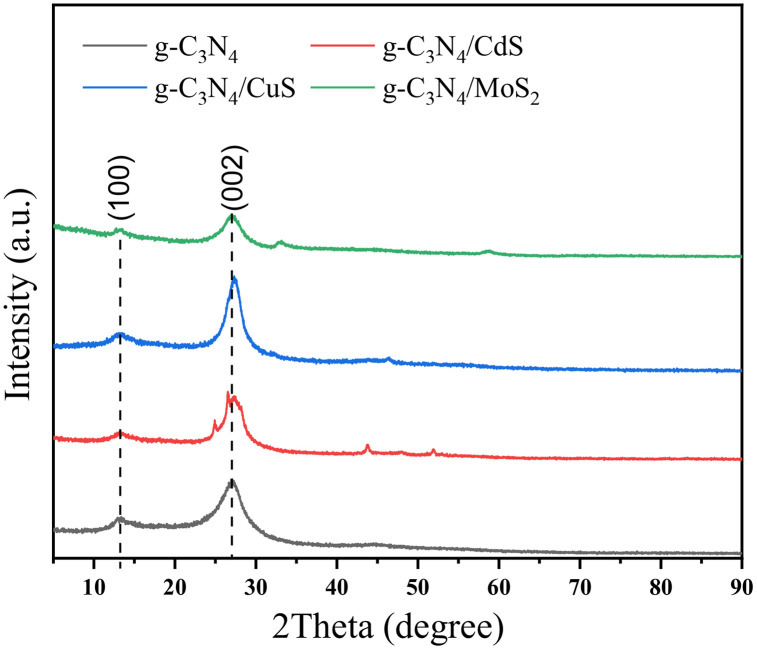
XRD spectra of carbon nitride composite material.

The characterization results of the crystal structure of the material are shown in Fig 4. The XRD pattern of g-C_3_N_4_ shows two distinct diffraction peaks at 12.8 ° and 27.6 °, corresponding to the (100) and (002) crystal planes of graphitic carbon nitride, respectively. The intensity of the above diffraction peaks on the XRD patterns of g-C_3_N_4_/CdS, g-C_3_N_4_/CuS, g-C_3_N_4_/MoS_2_ weakens, especially the diffraction peak at 2 θ = 12.8 °, indicating that the introduction of CdS, CuS, and MoS_2_ has a certain degree of influence on the van der Waals forces and π – π stacking forces between g-C_3_N_4_ graphite layers.

**Fig 5:**
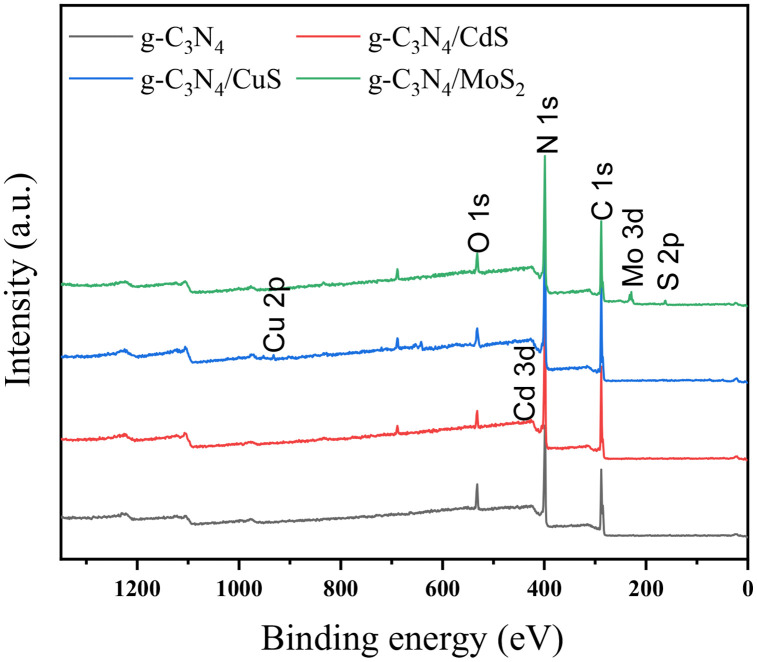
XPS full spectrum of carbon nitride composite material.

The XPS spectrum shown in Fig 5 confirms that g-C_3_N_4_ is mainly composed of C and N. The signals of Cd, Cu, Mo, and S elements can be observed in the subsequent reaction products, which are consistent with the composition of the prepared composite material.

#### Surface functional group analysis.

**Fig 6:**
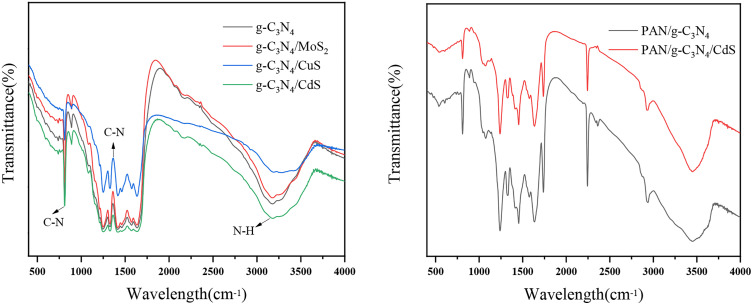
FT-IR spectra of g-C_3_N_4_, g-C_3_N_4_/MoS_2_, g-C_3_N_4_/CuS, g-C_3_N_4_/CdS, PAN/g-C_3_N_4_, PAN/g-C_3_N_4_/CdS.

The results of functional group characterization of the prepared composite photocatalysts using FT-IR are shown in Fig 6. The absorption peaks of FT-IR spectra of g-C_3_N_4_, g-C_3_N_4_/MoS_2_, g-C_3_N_4_/CuS, g-C_3_N_4_/CdS are basically the same, and all of them exhibit the typical g-C_3_N_4_ triazine ring structure. The narrow absorption peak at 810 cm^-1^ is caused by the bending vibration of C-N ring in triazine ring structure, the absorption peak at 1200 ~ 1600 cm^-1^ is caused by the telescopic vibration of C-N ring in g-C_3_N_4_ structure, and the absorption peak at 3000 ~ 3500 cm^-1^ is caused by the telescopic vibration of N-H in g-C_3_N_4_ structure [36]. Given that the infrared characteristic peaks of metal sulfides are generally below 500 cm^-1^, the characteristic peaks of the photocatalytic composites did not appear in the infrared maps.

Infrared spectral analysis of the two flexible polyacrylonitrile nanofibers showed that the two catalysts had obvious absorption peaks at 500 ~ 1750 cm^-1^, 2000 ~ 2250 cm^-1^, and 2750 ~ 3000 cm^-1^, which were superimposed on the characteristic peaks of PAN and g-C_3_N_4_. Among them, 810 cm^-1^ and 1200 ~ 1600 cm^-1^ are the characteristic peaks of g-C_3_N_4_, and 2240 cm^-1^, 2850 cm^-1^, etc. are the characteristic peaks of PAN matrix fibers, which indicate that g-C_3_N_4_-based photocatalysts have been successfully introduced into the structure of PAN fibers. Specifically, a sharp and strong peak at 2240 cm^-1^ is the telescopic vibrational absorption peak of cyano (C ≡ N), while a set of blunt absorption peaks near 2850 cm^-1^ corresponds to methyl (-CH_3_), and absorption peaks are also present at 3000 ~ 3500 cm^-1^, corresponding to O-H and N-H. Fig 6 also shows that the infrared spectra of both PAN/g-C_3_N_4_ and PAN/g-C_3_N_4_/CdS are basically consistent at the corresponding wavelengths. Analyzing the changes of each functional group indicates that the original structures of these two photocatalysts remain intact and the main properties are well preserved after the composite, which further confirms that the synthesis process has not caused any adverse effects on them.

#### Optical property analysis.

**Fig 7:**
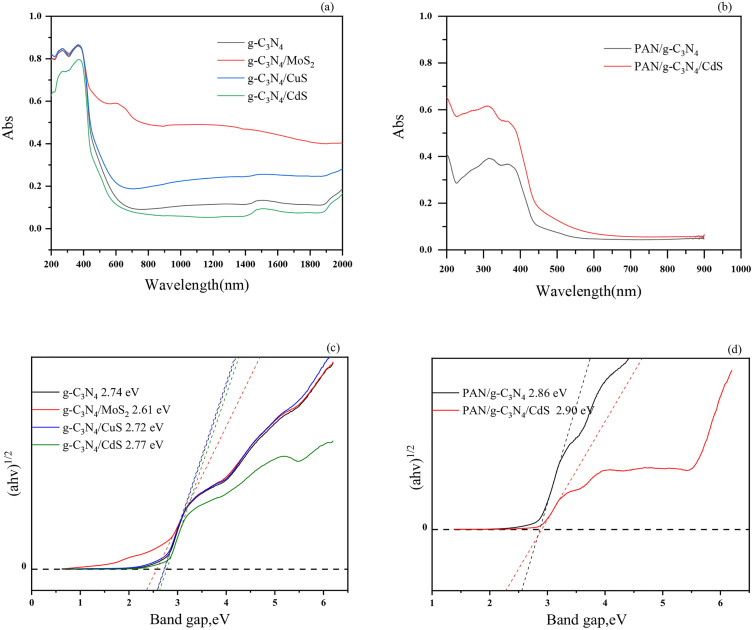
UV-VIS-NIR spectra of composite material (a, b) and relationships between (αhν)^1/2^ and band gap (hν) (c, d).

The light absorption range of composite photocatalyst was obtained using a UV-NIR spectrophotometer with a scanning range of 200–2000 nm, as shown in Fig 7. The carbon nitride photocatalytic material has high light absorption performance in the wavelength region below 460 nm, and the light absorption performance also shows a small increase in the near-infrared region, while it exhibits low light absorption performance in the visible region from 600 to 800 nm. g-C_3_N_4_/MoS_2_ photocatalytic materials exhibit strong light absorption performance in both the UV and NIR regions, and they also show good light absorption performance in the visible region. Efficient light absorption is essential for improving the performance of photocatalysts, which can significantly enhance the efficiency of photocatalysts.

The flexible polyacrylonitrile nanofiber material has the best light absorption performance in UV region below 400 nm, and also has a good light absorption performance in the visible region from 400 to 600 nm, and the worst light absorption ability in the infrared region above 760 nm. CdS introduction can effectively enhance the light absorption performance of flexible nanofiber materials in the visible region.

The band gap of the samples was further confirmed by using the Tauc/David–Mott model described by the equation [37]:


$${\left(\alpha \text{h}\nu \right)}^{\text{1}/\text{n}}=\text{A}(h\nu -{\text{E}}_{\text{g}})$$

where h is the Planck’s constant, ν is the frequency of vibration, α is absorption coefficient, Eg is the gap energy of the semiconductor and A is a proportional constant. For indirect bandgap semiconductors, the value of the exponent n is defined as 2 and the fitting results in Fig 7 show that the band gap of g-C_3_N_4_、g-C_3_N_4_/MoS_2_、g-C_3_N_4_/CuS、g-C_3_N_4_/CdS、PAN/g-C_3_N_4_、PAN/g-C_3_N_4_/CdS composite are approximately 2.74 eV, 2.61 eV, 2.72 eV, 2.77 eV, 2.86 eV and 2.90 eV, respectively. Compared with pure g-C_3_N_4_, the band gap of PAN/g-C_3_N_4_/CdS composite increase. Band gap widening is usually accompanied by an upward shift in the valence band position, and the hole oxidation ability is significantly enhanced.

### Analysis of photocatalytic degradation of SMT

#### Effect of LED wavelength on catalytic performance.

**Fig 8:**
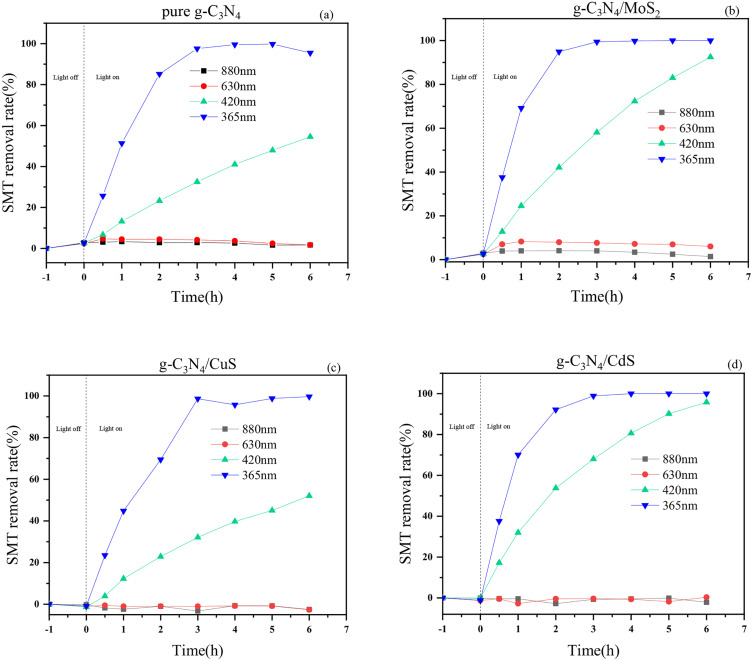
The influence of wavelength on SMT removal rate: (a) g-C_3_N_4_; (b)g-C_3_N_4_/MoS_2_; (c)g-C_3_N_4_/CuS; (d)g-C_3_N_4_/CdS.

The composite photocatalysts were driven to degrade sulfadimethoxine pyrimidine by LED monochromatic light at wavelengths of 880 nm, 630 nm, 420 nm and 365 nm, respectively, to obtain the removal rate of SMT by composite photocatalysts under the driving of different wavelengths, and the results are shown in Fig 8. SMT removal rate of g-C_3_N_4_ at wavelengths of 880 nm and 630 nm was very low after the light was turned on for 6 h; at wavelength of 420 nm, SMT removal rate was 54.55% after the light was turned on for 6 h; and at wavelength of 365 nm, SMT removal rate reached 95.51% after the light was turned on for 6 h. Compounding g-C_3_N_4_ with metal sulfides can effectively enhance the photocatalytic degradation efficiency of SMT. The photocatalyst g-C_3_N_4_/CuS showed a SMT removal rate of 52.06% at a wavelength of 420 nm after the light was turned on for 6 h, and 99.70% at a wavelength of 365 nm after the light was turned on for 6 h. SMT removal rate of the photocatalyst g-C_3_N_4_/MoS_2_ was only 1.43% at 880 nm and 6.03% at 630 nm after the light was turned on for 6 h; at the wavelength of 420 nm, SMT removal rate was 92.49% after the light was turned on for 6 h; at the wavelength of 365 nm, SMT removal rate reached 100.00% after the light was turned on for 6 h. Among the three composite photocatalysts, g-C_3_N_4_/CdS showed the best SMT removal effect. At the wavelength of 420 nm, SMT removal rate was 95.82% after the light was turned on for 6 h; at the wavelength of 365 nm, SMT removal rate was 92.16% after the light was turned on for 2 h, and then the removal rate reached 100.00% after 4 h. The removal rates of SMT by the four photocatalysts showed the same trend, i.e., the larger the LED wavelength, the lower the removal rate of SMT, which is detrimental to the catalytic performance of carbon nitride photocatalytic materials, especially at wavelengths higher than 630 nm, where the composites almost do not degrade SMT; on the contrary, the shorter the LED wavelength, the higher the photon energy provided, the more favorable it is to stimulate the carbon nitride photocatalytic material to generate electron leaps, which leads to the generation of active species such as holes, hydroxyl radicals, single-linear oxygen, and ultimately leads to the increase of SMT removal rate.

#### Effect of pH on catalytic performance.

**Fig 9:**
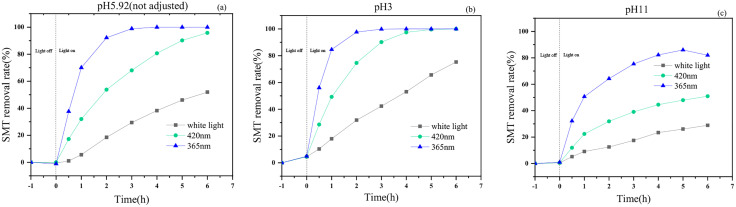
The effect of initial pH of solution on the photocatalytic performance of g-C_3_N_4_/CdS: (a)pH = 5.92; (b) pH = 3; (c) pH = 11.

Solution pH is also one of the important factors affecting the degradation of antibiotics. Take g-C_3_N_4_/CdS samples with the best removal effect on sulfadimethoxine pyrimidine, adjust the initial pH of the solution to 3 and 11, respectively, and carry out the photocatalytic performance experiments under the wavelengths of 365 nm, 420 nm, and white light, and compare the removal performance with that without adjusting the pH (pH = 5.92), to study the effect of the initial pH of the solution on the degradation of SMT by g-C_3_N_4_/CdS. As shown in Fig 9, the removal rate with white light drive for 6 h was 51.89% without pH adjustment; under the condition of wavelength 420 nm, the removal rate with light on for 6 h was 95.82%; and under the wavelength 365 nm, the removal rate with light on for 3 h reached 98.91%, and the removal rate with light on for 4 h reached 100%. Adjusting the pH to 3 promoted the catalytic degradation of sulfadimethylpyrimidine by g-C_3_N_4_/CdS, with a removal rate of 75.30% with white light drive for 6 h. The removal rate reached 100% with the light on for 6 h at a wavelength of 420 nm, and 99.80% with the light on for 3 h at a wavelength of 365 nm, and 100% for 4 h. Adjusting pH to 11, the photocatalytic degradation of SMT by g-C_3_N_4_/CdS was significantly inhibited, with a removal rate of 28.93% with white light drive for 6 h; 50.92% with the light on for 6 h at a wavelength of 420 nm; and 75.38% at a wavelength of 365 nm with the light on for 3 h, and only 81.96% for 6 h. The effect of solution pH on the photocatalytic performance of the composites mainly lies in the effect on the generation of active species. Under the acidic environment, the holes generated by photoexcitation can react with water molecules to generate hydroxyl radicals, and the appropriate amount of H^+^ helps the holes to be converted into radicals with strong oxidizing properties more effectively, thus promoting the photocatalytic performance; in alkaline environments, OH^-^ competes for adsorption with surface cavities and reduces the generation efficiency of active species on the one hand, and on the other hand, excess OH^-^ reacts with hydroxyl radicals to generate less active species (e.g., superoxide radicals), which leads to a significant decrease in SMT removal [38].

#### Catalytic properties and stability of flexible photocatalytic materials.

**Fig 10:**
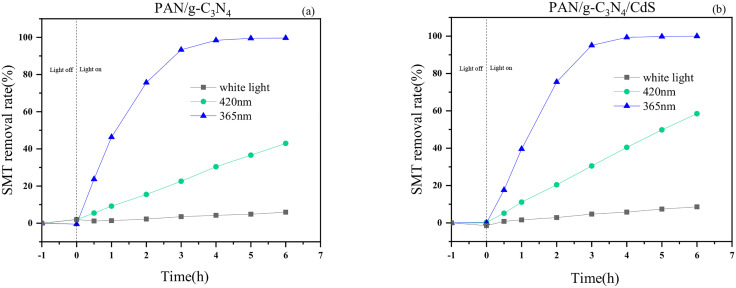
Photocatalytic performance of flexible nanofiber catalysts at different wavelengths.

Fig 10 shows the comparison of SMT removal by electrostatic spinning prepared PAN/g-C_3_N_4_ and PAN/g-C_3_N_4_/CdS at different wavelengths. As can be seen from Fig 10, neither PAN/g-C_3_N_4_ nor PAN/g-C_3_N_4_/CdS showed any decrease in antibiotic concentration within 1 h of dark adsorption, and there was no adsorption phenomenon. After the light was turned on, the removal rate of SMT gradually increased with the increase of light time, and the removal rate reached the highest after 6h of catalyzing. At the wavelength of 420 nm and after the light was turned on for 6 h, SMT removal rate of PAN/g-C_3_N_4_ was 42.92%, and SMT removal rate of PAN/g-C_3_N_4_/CdS was 58.45%; while at the wavelength of 365 nm and after the light was turned on for 6 h, SMT removal rate of PAN/g-C_3_N_4_ reached 99.65%, and that of PAN/g-C_3_N_4_/CdS reached 100.00%. PAN/g-C_3_N_4_ and PAN/g-C_3_N_4_/CdS also showed certain removal effect of SMT under white light, and SMT removal rate was 5.93% for PAN/g-C_3_N_4_ and 8.56% for PAN/g-C_3_N_4_/CdS after the light was turned on for 6 h. Therefore, the longer the LED wavelength, the worse the effect of degrading antibiotics, and conversely, the shorter the LED wavelength, the higher the removal efficiency and the better the effect of the photocatalyst on antibiotics. The above results show that the flexible nanofiber photocatalytic material prepared in this study can achieve excellent removal effect of SMT at 365 nm.

**Fig 11:**
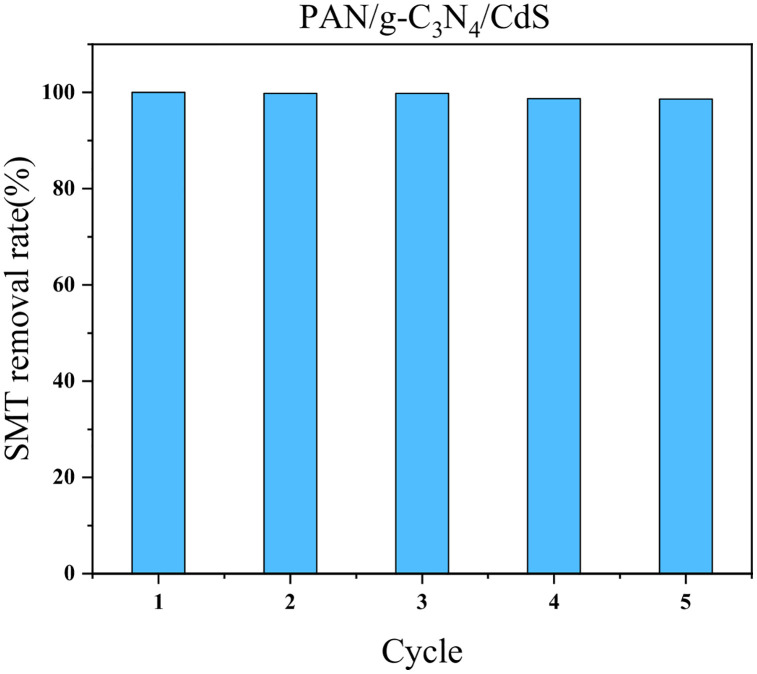
The cyclic performance of flexible nanofiber catalysts.

The stability and repeatability of photocatalytic materials are important parameters for their practical applications. Therefore, PAN/g-C_3_N_4_/CdS was selected to study the stability of the material in photocatalytic removal of SMT. The results are shown in Fig 11. Under the same experimental conditions, the material was subjected to 5 cycles of catalytic testing. After each cycle, the sample was washed with anhydrous ethanol to remove surface residues, and then dried in a vacuum oven at 60°C. After 5 cycles, the degradation rate of SMT by PAN/g-C_3_N_4_/CdS at a wavelength of 365 nm was 98.6%, indicating good stability for repeated use.

#### Mechanism of photocatalytic degradation of SMT.

**Fig 12:**
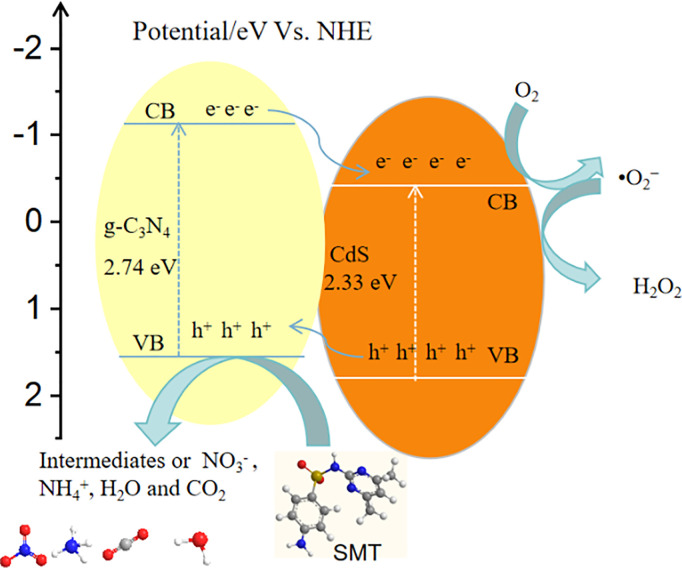
Photocatalytic mechanism scheme of as-prepared g-C_3_N_4_/CdS.


Fig 12 depicts a schematic diagram of the photocatalytic degradation of SMT over g-C_3_N_4_/CdS composite. The conduction band (CB) edge potential of g-C_3_N_4_ at −1.14eV is more negative than that of CdS at −0.44 eV, whereas the valence band (VB) edge potential of CdS at +1.69 eV is more positive than that of g-C_3_N_4_ at +1.60 eV [39,40]. Obviously, both g-C_3_N_4_ (band gap: 2.74 eV) and CdS (band gap: 2.33 eV) can absorb visible-light to produce photogenerated electron–hole pairs. The appropriate band potentials of the individual semiconductors favor the spontaneous transfer of the photogenerated electrons from the g-C_3_N_4_ surfaces to CdS, and the photogenerated holes from the CdS surfaces to g-C_3_N_4_. In other words, photogenerated electrons and holes move in opposite directions, which effectively reduce the recombination probability and enhance the charge separation efficiency. Especially, the photogenerated holes rapidly transferring to the solution leave not enough holes on CdS to cause photocorrosion. Subsequently, as the photogenerated electrons located on the CB of g-C_3_N_4_ have a more negative potential (−0.44 eV) than the standard redox potential of O_2_/•O_2_^−^ −(−0.33 V vs NHE), they can react with oxygen to form •O_2_^−^. As the potential of •OH/H_2_O (2.68 V vs NHE) is more positive than the VB of g-C_3_N_4_ (1.57 eV), the photo-generated holes located on the VB of CdS cannot react with H_2_O to obtain •OH, but can directly react with organic pollutants on the surface of the catalysts to form CO_2_, H_2_O or other small inorganic molecules. In addition, the unique ultrathin nanosheet structure of g-C_3_N_4_ improves the photocatalytic performance. The reason is that the ultrathin nanosheet structure can effectively shorten the distance for the migration of photogenerated carriers to the interfaces, which reduces the recombination probability of photo-generated carriers. According to the above results, the transport and degradation mechanism of the photo-generated carrier of g-C_3_N_4_/CdS composite photocatalyst can be described by the following equation:

$$g-C_{\text{3}}N_{\text{4}}+hv\to g-C_{\text{3}}N_{\text{4}}(e^{-})+g-C_{\text{3}}N_{\text{4}}(h^{+}\;)$$

$$CdS+hv\to CdS(e^{-})+Cds(h^{+}\;)$$

$$e^{-}+O_{\text{2}}\to \cdot O_{\text{2}}^{-}$$

$$\cdot O_{\text{2}}^{-}+H^{+}\to {HO}_{\text{2}}^{\cdot }$$

$${HO}_{\text{2}}^{\cdot }+H^{+}+e^{-}\to H_{\text{2}}O_{\text{2}}$$

$$\cdot O_{\text{2}}^{-},H_{\text{2}}O_{\text{2}},h^{+}+SMT=\;degradation\;products$$

## Conclusion

(1) g-C_3_N_4_, g-C_3_N_4_/MoS_2_, g-C_3_N_4_/CuS, and g-C_3_N_4_/CdS photocatalysts were prepared by thermal polymerization. The physical and chemical properties of the photocatalytic materials, such as functional groups and surface morphology, were characterized by means of SEM, FT-IR, and UV-VIS-NIR, and the results showed that the prepared composite photocatalytic materials did not change the morphological structure of g-C_3_N_4_; carbon nitride photocatalytic materials have high light absorption performance in the wavelength region below 460 nm, and the light absorption performance also shows a small increase in the near-infrared region, while they exhibit low light absorption performance in the visible region from 600 to 800 nm. The introduction of metal sulfides can effectively enhance the light absorption performance and photocatalytic activity of the composites.
(2) Among the four photocatalytic materials, g-C_3_N_4_, g-C_3_N_4_/MoS_2_, g-C_3_N_4_/CuS, g-C_3_N_4_/CdS, g-C_3_N_4_/CdS composite photocatalytic material showed the best removal of sulfadimethylpyrimidine and the most stable photocatalytic performance. The complete removal of SMT could be achieved after photocatalysis for 6 h at both wavelengths of 420 nm and 365 nm. The initial pH of the solution is also one of the important factors affecting the degradation of antibiotics. Under acidic environment, it was favorable to promote the removal of sulfadimethylpyrimidine by carbon nitride composite photocatalytic materials and showed different photocatalytic activities under strongly and moderately acidic conditions. At the wavelength of 365 nm, the removal rate reached 84.61% with the light on for 1 h at pH 3, while the removal rate was 70.05% with the light on for 1 h without adjustment. g-C_3_N_4_/CdS had the highest photocatalytic activity at pH = 3.
(3) The enhanced photocatalytic activity of g-C_3_N_4_/CdS is mainly attributed to the synergistic effect between g-C_3_N_4_ and cadmium sulfide. This synergistic effect can significantly reduce the probability of photogenerated electron-hole pairs recombination, improve the charge separation efficiency, and thus enhance the photocatalytic performance.
(4) Flexible polyacrylonitrile carrying carbon nitride nanofiber photocatalysts PAN/g-C_3_N_4_ and PAN/g-C_3_N_4_/CdS were prepared by electrostatic spinning technique, and the photocatalytic materials were successfully carried on the fiber by characterizing the physical and chemical properties of the photocatalysts. Optical property analysis shows that PAN/g-C_3_N_4_/CdS composite photocatalytic materials have good light absorption properties in UV and visible regions.
(5) The flexible nanofiber photocatalytic material can achieve excellent removal of SMT at 365 nm. The SMT removal rate of PAN/g-C_3_N_4_ reached 99.65% and that of PAN/g-C_3_N_4_/CdS reached 100.00% after the light was turned on for 6 h under the wavelength of 365 nm.
